# An analysis of the annual mobility of Polish Konik horses depending on habitat, season, and time of the day

**DOI:** 10.5194/aab-65-239-2022

**Published:** 2022-07-12

**Authors:** Ryszard Pikuła, Daniel Zaborski, Wilhelm Grzesiak, Mirosław Smugała

**Affiliations:** 1 Laboratory of Horse Breeding and Animal Assisted Therapy, West Pomeranian University of Technology, 71-270 Szczecin, Poland; 2 Department of Ruminants Science, West Pomeranian University of Technology, 71-270 Szczecin, Poland

## Abstract

The aim of the present study was to analyse the mobility of Polish Konik horses in their natural environment. The study was conducted on a herd of 15 Polish Konik horses in 2018. The Global Positioning System (GPS) transmitter was used to track the horses' movements. Two habitats (forest and meadows), four seasons (autumn, winter, spring, and summer), and four times of the day (morning, midday, evening, and night) were distinguished. Season, habitat, and time of the day as well as the interaction among them significantly ( p<0.0001 ) affected the mobility of Polish Konik horses. The use of the GPS device enabled tracking of horses' mobility also at night, which made the results more complete compared with other similar studies.

## 1 Introduction

Polish Konik is the only primitive horse breed originating directly from wild tarpans (*Equus caballus* *gmelini Ant*.) (Kownacki, 1995). Tarpans are considered by many authors (Vetulani, 1933; Zwolinski, 1976) to be the original wild form, i.e. a distant ancestor of many horse breeds. Others state that tarpans were feral domestic horses (Czapski, 1874). The Polish Konik breed was mainly formed under natural environmental conditions. Therefore, it retained many traits of primitive horses, which allows researchers to study the natural behaviour of these animals.

In order to preserve the breed and its primitive traits, Polish Konik horses are reserve-bred, which involves free-range rearing, the lack of human intervention, and exposing the horses to natural selection factors (PZHK, 2020). Researchers have aimed at understanding the biology of Polish Konik for years. Kownacki et al. (1978) performed 24 h observation of Polish Konik herds, but only the application of modern research methods such as the Global Positioning System (GPS) has made it possible to fully characterize the migration patterns and locomotor activity of free-living Polish Konik horses.

The need of horses, formed during evolution, is movement, which, under natural conditions, is mainly stimulated by the satisfaction of hunger and thirst, migration, or escape from danger. It also determines social contacts and herd hierarchy (Rehm, 1981). Lack of movement not only results in a series of problems associated with the locomotor system (such as tendon weakness, reduced bone density, and abnormalities in the hoof structure and mechanics) (Porr et al., 1997; Nielsen et al., 2000), but also negatively affects the circulatory and respiratory systems (Vervuert and Coenen, 2002).

Polish Konik horses have also been used in nature reserves created to replicate Europe's prehistoric past, i.e. to “rewild” the area, by populating it with the kinds of animals (e.g. tarpans) that lived there many thousands of years ago. For instance, the population of Konik horses was acquired from Poland in the 1980s and used as “proxies” for extinct tarpans in the 6000 ha Oostvaardersplassen nature reserve in the Netherlands. Konik horses, Heck cattle, and bison were also introduced to a similar 5700 ha nature reserve, located near Lake Pape in Latvia (Marris, 2009). Therefore, reserve breeding is of economic significance to active environmental protection, and Polish Konik horses constitute a valuable source of genetic resources.

Finally, the improvements in spatial and temporal data resolution, reduced device size, and increased battery longevity made GPS technology suitable for the analysis of horse movements over a continuous period of time and not only over a certain part of the day (Hampson et al., 2013; Krueger et al., 2014; Hennig et al., 2018; Walden-Schreiner et al., 2018). Studies on Polish Konik horses which are kept in a stable or reserve system provide information about the natural behaviour of horses as well as their greater healthiness in comparison with animals maintained under artificial conditions created by man. Despite seasonal food shortages, Konik horses are granted the so-called “five freedoms” of well-being, which is confirmed by their good health, longevity (up to 37 years), high reproductive performance, and social relations. Therefore, the understanding of the behaviour of free-living horses is the basis for establishing modern stabling systems. The present study focuses on locomotor activity as a factor that played a major role in the evolution of Equidae. Similar conditions should be created for modern horses, which are open-space animals that feed themselves in motion (Salau et al., 2020). Free-roaming horses cover a daily distance of about 6 km, whereas those housed in box stalls cover only 800 to 900 steps. One example of the practical application of this knowledge is the so-called paddock paradise system (Jackson, 2006).

Therefore, the aim of the present study was to analyse the seasonal mobility of Polish Konik horses in their natural environment using GPS technology.

## 2 Materials and methods

**Figure 1:**
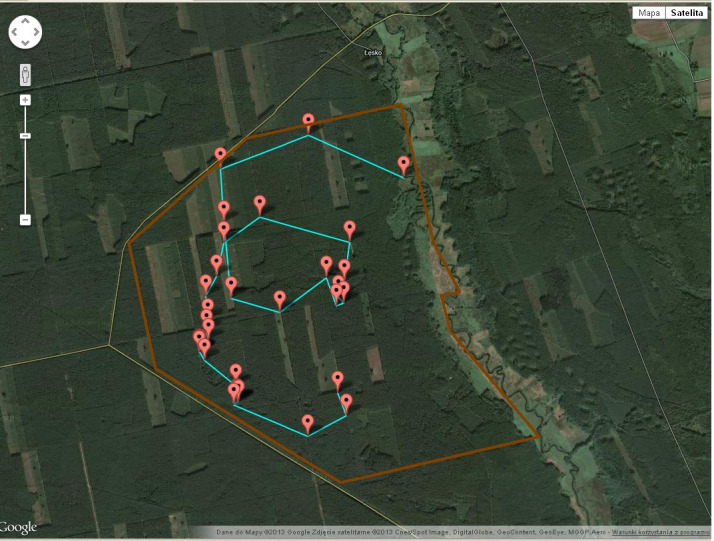
An example of the tracks used by the horses within the nature reserve area. Map data © 2013 Google, Satellite image © 2013 Cnes/Spot Image, DigitalGlobe, GeoContent, GeoEye, MGGP Aero.

The study on the distance covered by Polish Konik horses was conducted in a forest and meadow area of 700 ha in the Zagroda breeding reserve, which is located in the Kliniska Forest District near Szczecin. The observations were carried out between 2018 and 2019 using the Global Positioning System (GPS) transmitter (Ecotone, Gdynia, Poland) attached to a broodmare (the so-called wolf collar) from a herd of 15 horses led by the stallion Nagaj. The device sent the data on herd location every hour, which was the basis for determining the distance covered by horses during 1 h. The distance was expressed in metres in the so-called straight line. An example of the tracks used by the horses within the nature reserve area is shown in Fig. 1. During the study period, there were two herds, but the second one was under formation (in a different part of the nature reserve located about 8 km from the territory of the studied herd) and consisted of several horses. Therefore, interactions between herds did not occur, so they did not affect mobility. No other interactions were found during the study period; however, they cannot be completely excluded. The horses were additionally fed with hay during winter. The hay was provided in the area between the meadow and the forest in December, January, and February, practically every second day. The methods were described in detail in our previous work (Pikuła et al., 2020).

Two basic habitats were distinguished in the present study:

1. forest, including the area of old-growth forest, coppice, forest plantations, fire lanes, and clearings, and
2. meadows, including the area of meadows (approximately 70 ha) located along the Ina River.

To investigate the spatial and temporal trends in the migration of Polish Konik horses within the habitats, Ecotone software was used. The distance was recorded for 24 consecutive hours (over the period of 30 d during each season), which were grouped into the following times of the day:

1. morning, from 06:00 to 12:00 local time;
2. midday, from 12:00 to 18:00 local time;
3. evening, from 18:00 to 24:00 local time;
4. night, from 00:00 to 06:00 local time.

In addition, season was taken into account in the study, i.e. autumn (the measurements collected in October), winter (the measurements collected in January), spring (the measurements collected between April and May), and summer (the measurements collected in July).

The data were analysed using the following model:

1 $$\begin{array}{rl} y_{ijkl} & =\mu +a_{i}+b_{j}+c_{k}+ab_{ij}+bc_{jk}\\ & +abc_{ijk}+\beta \left(x_{ij}-\bar{x}\right)+e_{ijkl}, \end{array}$$

where $y_{ijkl}$ is the value of the dependent variable; μ is the overall mean; $a_{i}$ is the effect of time of the day; $b_{j}$ is the effect of season; $c_{k}$ is the effect of habitat; $ab_{ij}$ is the effect of the interaction between the time of the day and season; $ac_{ik}$ is the effect of the interaction between the time of the day and habitat; $bc_{jk}$ is the effect of the interaction between season and habitat; $abc_{ijk}$ is the effect of the interaction among the time of the day, season and habitat; β is the regression coefficient; $x_{ij}$ is the value of the co-variate (mean daily temperature); $\bar{x}$ is the overall mean of the covariate; and $e_{ijkl}$ is the random error.

Statistical analysis was carried out using Statistica 13.1 software (Dell Inc., Tulsa, OK, USA). The graphs were prepared using Microsoft Excel (Redmond, Washington, USA).

## 3 Results

An analysis of the distances covered annually by Polish Konik horses revealed a statistically significant effect of season, habitat, time of the day, and their interaction ( p<0.0000 ) on locomotor activity (Table 1). Only temperature did not significantly affect the distance covered by horses.

**Table 1:** The values of the F test for factors included in the model.

Effect	F	P
Intercept	307.32	0.0000
Habitat	320.59	0.0000
Season	84.20	0.0000
Time of the day	30.62	0.0000
Temperature	0.12	0.7297
Season and habitat	16.42	0.0000
Season and time of the day	7.43	0.0000
Habitat and time of the day	8.24	0.0000
Season, time of the day, and habitat	5.81	0.0000

It can be noticed that horses covered the longest average distance in the forest (239 m h ${}^{-1}$ ) and the shortest one in the meadow habitat (about 96 m h ${}^{-1}$ ) (Table 2).

**Table 2:** The mean distance (m h ${}^{-1}$ ) covered by horses depending on habitat, time of the day, and season.

	n	Mean ${}^{*}$	SE
Habitat			
Meadow	1585	96.10 ${}^{A}$	2.76
Forest	1367	239.30 ${}^{B}$	6.36
Time of the day			
Night	738	123.95 ${}^{A}$	5.44
Morning	738	179.65 ${}^{B}$	6.08
Midday	738	216.47 ${}^{C}$	8.88
Evening	738	127.06 ${}^{\mathrm{AD}}$	6.87
Season			
Winter	720	165.60 ${}^{A}$	7.15
Spring	744	191.89 ${}^{B}$	7.02
Summer	744	218.53 ${}^{C}$	7.72
Autumn	744	71.23 ${}^{D}$	4.84

${}^{A,\;B,\;C}$ Values within columns marked with different superscripts are significantly different at p<0.01 . ${}^{*}$ The distance travelled per day and month cannot be directly calculated by multiplying the distance travelled in m h ${}^{-1}$ by 24 or 720 h. The distance calculated in this way would only be a general approximation, since horses travelled different distances each day and each hour.

**Table 3:** Total time during which horses remained in different habitats and the frequency of habitat change during the day.

Season	Forest	Meadow	Mean number	Standard	Mode	Min	Max	Total number
	(h)	(h)	of habitat	error				of changes
			changes per day					per month
Winter	367	353	5.17	2.42	7	1	11	155
Spring	268	476	4.50	2.49	7	1	10	135
Summer	467	277	4.09	1.68	3	1	9	127
Autumn	252	492	3.45	1.23	3	2	7	107

The number of hours per month during which horses remained in different habitats is presented in Table 3. Horses spent more time in the forest habitat in winter and, especially, in summer (50.97 % and 62.77 % of the total time, respectively), whereas they spent more time in the meadow habitat in spring and autumn (63.98 % and 66.13 % of the total time, respectively) (Table 3). Horses changed habitat most frequently in winter and spring (seven times during the day; 5.17 and 4.50 times on average, respectively) and least frequently in summer and autumn (three times during the day; 4.09 and 3.45 times on average, respectively). The total number of habitat changes in different months ranged between 107 in autumn and 155 in winter (Table 3).

**Figure 2:**
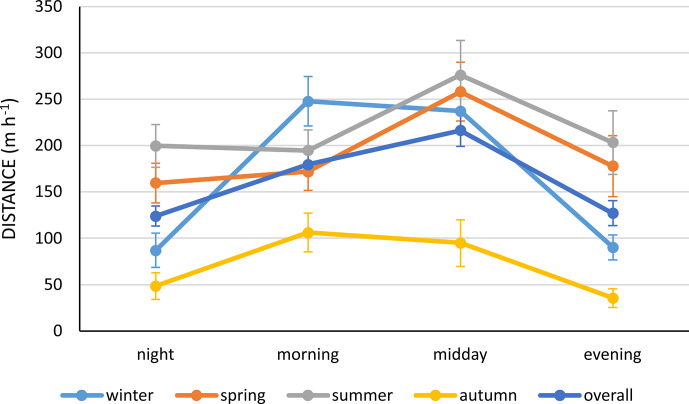
The mean distance (m h ${}^{-1}$ ) travelled by Polish Konik horses during different times of the day and seasons.

In the whole year, horses covered the longest average distance during the midday hours (from 09:00 to 15:00 local time). The average locomotor activity of Polish Konik horses increased until the midday hours and decreased subsequently (Fig. 2).

However, one should notice the differences among individual seasons (the interaction season and the time of the day) (Fig. 2). In general, the locomotor activity increased during the midday hours in spring and summer, whereas it increased already during the morning hours and decreased in the evening in winter. In autumn, activity slightly increased during the morning and midday hours (approximately 50 to 100 m h ${}^{-1}$ ) and decreased subsequently.

**Figure 3:**
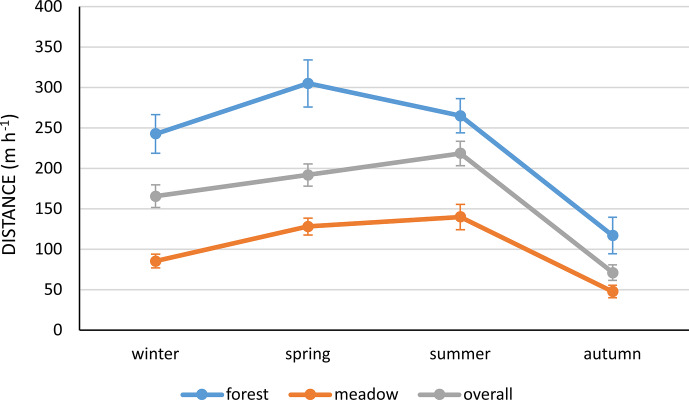
The mean distance (m h ${}^{-1}$ ) travelled by Polish Konik horses during different seasons in both habitats.

**Figure 4:**
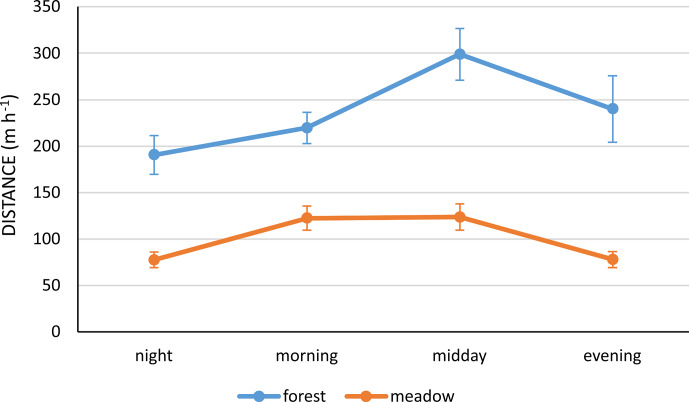
The mean distance (m h ${}^{-1}$ ) travelled by Polish Konik horses during different times of the day in both habitats.

The highest locomotor activity was recorded in summer (218.5 m h ${}^{-1}$ ) and the lowest in autumn (71.2 m h ${}^{-1}$ ). In winter, horses travelled 165 m h ${}^{-1}$ on average and in spring about 192 m h ${}^{-1}$ on average (Table 2 and Fig. 3). At different times of the day, horses covered the longest distance in the forest habitat (200–300 m h ${}^{-1}$ ) and a markedly lower one in the meadow habitat (Fig. 4).

**Figure 5:**
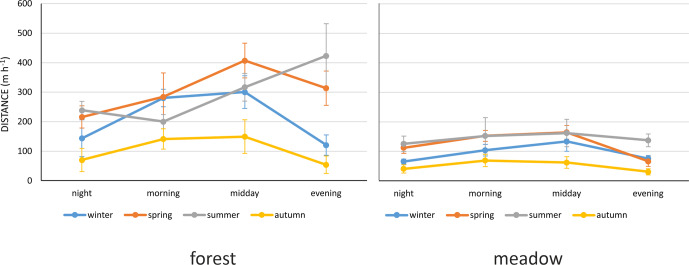
The mean distance (m h ${}^{-1}$ ) travelled by Polish Konik horses during different times of the day and seasons in both habitats.

However, greater differences were observed in the forest distances than in the meadow ones. The interaction between season and habitat showed that, in the meadows, locomotor activity slightly increased from winter to summer and decreased subsequently. In the forest, locomotor activity increased from winter to spring and decreased slightly in summer. It fell further in autumn (Fig. 3). The three-way interaction between season, time of the day, and habitat was also statistically significant, which resulted in some differences in locomotor activity depending on season and habitat, especially in the forest (Fig. 5).

## 4 Discussion

It can be stated that one of the factors affecting an increased locomotor activity of Polish Konik horses in the present study was the availability of the feed base. They travelled the longest distance in search of food in the forest, i.e. 239 m h ${}^{-1}$ on average (5.7 km d ${}^{-1}$ ). This distance differed significantly ( p<0.0000 ) from that covered in the meadow (96 m h ${}^{-1}$ on average, approximately 2.3 km d ${}^{-1}$ ). The changes in environmental conditions under which the horses lived were also caused by weather changes in different seasons, protection against sun exposure, and uncomfortable wind conditions. They rarely stayed in the forest for the whole day (more often in winter) or in the meadow (more often in spring or summer). Also, a significant difference in the distance travelled by Polish Konik horses among seasons could have resulted from general weather conditions and the fact that the horses were additionally fed with hay (starting in autumn) and were not forced to move in search of the feed base, like in the warmer periods. Unfortunately, this was human interference which limited the natural need for food search and reduced mobility. Statistically significant differences in the locomotor activity of Polish Konik horses between times of the day were noticed in the present study. They covered the shortest distance during the evening and night hours (approximately 127 and 124 m h ${}^{-1}$ , respectively; a total distance of about 750 m). In the remaining times of the day, the distances were approximately 180 and 216 m (i.e. a total distance of 1.0 to 1.3 km) (Table 2). In general, the locomotor activity increased until midday and decreased in the evening and at night. It should be mentioned that the monitoring of stock behaviour, especially when released to graze overnight, is rather rare (Walden-Schreiner et al., 2018). During the study period, interactions between herds of horses did not occur. No other interactions were found during this time; however, they cannot be completely excluded. Sporadically, increased mobility occurred during the night period, probably caused by the appearance of predators (such as wolves). A combined effect of season and time of the day, i.e. increasing daylight, on the activity of Polish Konik horses was also observed in the present study, which was confirmed by a statistically significant interaction (Fig. 2). In autumn and winter, the decrease in activity already occurred in the midday and evening hours (this probably resulted from replenishing feeding racks with hay), whereas in spring and summer, horses were still active at this time. The only factor analysed in the present study whose effect turned out to be non-significant was temperature. This finding is in contrast to that obtained by Claudi and Hoy (2013), who reported a significant effect of temperature on daily distances. At temperatures below 10 ${}^{\circ }$ C, horses moved on average 6722 m each day, and, at temperatures above 10 ${}^{\circ }$ C, they covered a longer daily distance of 9840 m. The effect of different factors affecting horse mobility identified in the present and other studies is summarized in Table 4.

**Table 4:** Reasons for mobility in different horse breeds.

Reason	Comment	Reference
Feed	Konik horses travelled the longest distance in the forest. The distance covered in the meadow and hay was significantly shorter.	Pikuła et al. (2020)
	Mature standardbred mares on the pasture treatment spent a greater amount of time in motion than those on the hay treatment. Horses also travelled a greater distance on pasture than on hay. Horses on hay travelled a greater distance per unit area than those on pasture. The mean speed for horses maintained on hay was greater than that for animals kept on pasture, whereas the time in motion remained greater in pasture compared with hay.	Weinert et al. (2020)
Season	Polish Konik horses from the Popielno Reserve most often moved in winter and least often in autumn. Horses had to search for feed during winter and for shelter from insects in summer, whereas stallions covered longer distances in spring due to increased sexual activity associated with searching for new mates.	Golonka (2009)
	Biłgoraj horses from the Roztocze National Park and the Janowskie Forests Landscape Park spent more time in motion than did Polish Konik horses in winter and summer (Biłgoraj horses are influenced by noble breeds that are much more temperamental than other breeds and types).	Kapron et al. (2000)
	Adult Polish Konik horses, 2-year-old horses, and foals from the Roztocze National Park spent less time in motion in July compared with September.	Pluta et al. (2013)
	A statistically significant effect of season on the locomotor activity of Shetland pony mares was found.	Brinkmann et al. (2012)
	Horses of different breeds from Schleswig-Holstein, Germany, walked a greater distance in summer, during which they had the possibility to go to the pasture. Lower distances could be seen in winter.	Hildebrandt et al. (2020)
Time of the day	Konik horses covered the longest distance in the midday and evening hours. Similar distances were recorded in the evening and night as well as night and morning hours. Horse activity increased with the onset of the day.	Pikuła et al. (2020)
	Polish Konik horses were more active between 18:00 and 24:00 and between 06:00 and 12:00 local time, in both winter and summer. Polish Konik and Biłgoraj horses were more active during the day than the night in both seasons.	Kapron et al. (2000)
	Polish Konik horses of all age categories were motorically more active in July during the first half of the day. The greatest difference was observed for adult horses, and a smaller difference was observed for the remaining age categories. In September, adult horses and foals were slightly more active in the afternoon, whereas 2-year-old horses were more active in the morning, like in July.	Pluta et al. (2013)
	During the 2 h intervals in the morning of the same day, 50 cliques with more than six loose-housed horses were observed between 06:00 and 07:00 UTC. A total of 181 cliques were found between 08:00 and 09:00 UTC, with the largest one containing 12 horses. The widest interquartile ranges and the highest means in the number of cliques with more than six horses were observed between 08:00 and 09:00 UTC as well as between 16:00 and 17:00 UTC, while this metric behaved relatively stably outside these time periods. The size of the largest clique showed the widest interquartile range and the highest mean between 07:00 and 08:00 UTC. This metric was more variable over the day compared with the number of cliques and showed wide interquartile ranges and high means between 18:00 and 23:00 UTC. A significant effect of the grouping by hours on the clique metrics as well as density and diameter was found, whereas the grouping by days had a significant effect on the clique metrics but not on their density or diameter. The differences in the number and size of cliques as well as the density and diameter of the hourly networks during the day resulted from the main resting phase in northern Germany in June, which coincides with the absence of daylight. Another reason for an increased number of contacts and the forming of cliques was the opening of additional pasture in the boarding facility.	Salau et al. (2020)
Others	Walking distance travelled by horses from 28 farms located in Germany decreased by 1.4 % h ${}^{-1}$ of grazing time. Walking time and walking distance were the greatest for horses kept in box-stall stables and the smallest for those in free-range stables. Walking distance tended to increase with pasture size. Walking time of mares was significantly greater than that of geldings.	Schmitz et al. (2020)

In general, four keeping systems can be distinguished in Poland: a stable system, which is unrecommended since horses stay only in a stable; a stable–grazing system, in which horses stay on pasture during the day over the season and have access to paddocks after returning to the stable; a grazing system, in which horses remain on pasture all the time; and a cultural-herd system, in which horses stay in an open area throughout the year, supported by humans only under harsh climatic conditions, especially in winter (Pruski et al., 2006). Reserve breeding can be classified into this last method. It is of economic significance to active environmental protection, which requires knowledge of animal behaviour (both wild and domestic; Walden-Schreiner et al., 2018) and is applied not only for primitive horses but also in the extensive breeding of noble horses. Slaughter horses can also be kept in nature reserves. In addition, reserve breeding is used in different types of areas, such as forests (e.g. Białowieża Forest, Popielno) and lowlands (e.g. meadows in Czarnocin near Szczecin Lagoon).

The Polish Konik is a breed of primitive horses, which has its own main herd book. As already mentioned, horses are kept in a stable or reserve system. Herd surplus from reserve breeding is often sold and used as regular horses. The analysis and comparison of keeping methods in the horses of the same breed provide valuable information about changes that should be introduced to stable groups. The understanding of the behaviour of free-living horses is also the basis for appropriate stable keeping and its modernization. Studies on Polish Konik horses indicate an increased role of mobility in stable keeping systems (e.g. the so-called paddock paradise). Moreover, natural behaviour is always an adequate determinant of horse needs in relation to artificial conditions created by man.

In the study by Stanley et al. (2018), semiferal horses showed social stability regarding clique structure and individual network positions. Consequently, the analysis of the evolving contact structure of an inhomogeneous herd of domestic horses which are given the opportunity to move freely and fulfil their need to interact with their conspecifics may be of great interest to researchers studying animal behaviour (Salau et al., 2020). Krueger et al. (2014) identified two main factors affecting the initiation of movement in groups of feral horses using a visual observation method: herding (exclusive to alpha males) and departure (possible for any group member). Social bonds, the number of animals interacted with, and the spatial position did not significantly affect movement initiation. The authors also found a limited form of distributed leadership, with higher-ranking animals being followed more often.

Hampson et al. (2010a) studied the movement patterns of domestic Quarter Horse × Australian Stock Horses, Quarter Horses, and feral horses using GPS. Mean daily distances travelled by domestic horses were, in general, greater in larger paddocks (4.7, 6.1, and 7.2 km for 0.4, 4.0, and 16.0 ha, respectively). Feral horses, living in a 4000 ha paddock, covered the greatest daily distance (17.9 km), whereas those kept in the 6 m × 6 m yard travelled only 1.1 km. In their next study (Hampson et al., 2010b) on feral horses in “outback” Australia, a mean daily distance for all animals was 15.9 km (16.8 and 14.7 km for central Queensland horses and central Australian horses, respectively, but the difference was non-significant). Hampson et al. (2011) also assessed the ability of feral *Equus caballus* mares to cope in a novel feral environment using GPS. The mares taken from a semi-arid desert remained in good health but changed their movement behaviour when introduced to prime grazing habitat, whereas most mares captured from the prime grazing habitat and released in the semi-arid desert habitat died due to stress or starvation. The mares relocated to semi-arid desert did not easily adapt to relocation and had difficulties in taking up the movement strategy of local horses, which required long distance treks from a water hole to feeding areas. According to Hampson et al. (2011), the movement behaviour, range use, and health consequences of relocating equids may be of great importance to wildlife ecologists, animal behaviourists, and horse welfare groups engaged in relocating domestic or native horses to novel habitats. The study on Przewalski's horses was carried out in Mongolia by Kaczensky et al. (2008) using the ARGOS and GPS systems. It showed that the average daily straight-line distance between the consecutive days of observation was 3.5 km. An effect of season on the mean distance to the nearest water source was found (10.4 and 6.9 km in winter and summer, respectively), which shows that availability of water is an important factor determining space and habitat use for these animals. The analysis of the ranges of Przewalski's horses in the Gobi Desert revealed that the zoo-born animals are able to adapt their spatial use to the local habitat conditions; however their re-introduction to the area can be problematic due to the still-existing factors that caused their extinction such as competition with livestock for steppe habitats with sufficient water supply and interbreeding with domestic horses. Daily and weekly movement distances covered by feral horses in the different states of the USA were analysed by Hennig et al. (2018) using collars with GPS transmitters. The mean daily and weekly values were 9.0 and 62.1 km, respectively. The authors also provided information on the location of horses based on GPS data, which can be helpful in determining their impact on habitats occupied by other species. The GPS was also used in the study on horse-influenced habitat alteration and the correlation between utilization distributions generated from feral horses and dung pile density (Hennig et al., 2021). According to the authors, utilization distributions were a poor predictor of cumulative horse use, and additional management actions regarding feral horse use are needed to sustain high-quality habitat occupied by other species. In their subsequent study on the influence of digestive morphology and feeding strategy on the movement syndromes of feral horses in an arid-cold steppe of North America, Hennig et al. (2021) found that the animals exhibited more sedentary movements largely driven by selection for high biomass patches and areas closer to water.

An effect of the physiological status of dairy mares on their daily distance was investigated in Mongolia by Bat-Oyun et al. (2018) using the GPS method. The daily cumulative distance (approximately 0.6 to 1.0 km h ${}^{-1}$ ) and the daily maximum linear distance (about 3.1 to 3.8 km) differed significantly between the milking and non-milking periods. Finally, Claudi and Hoy (2013) found large individual differences in a daily distance among horses of different breeds using the GPS method. The distance (from 5.1 to 10.3 km) was not affected by age or sex, but breed had a significant effect on it (9.5, 8.1, and 7.7 km for Friesian horses, warmblood horses, and Connemara ponies, respectively). In the technical context of locomotor activity monitoring, Walden-Schreiner et al. (2018) designed an integrated system of behavioural analysis merging direct observation (frequently used for validating sensor-derived behaviour classification) and GPS data. The system was subsequently applied to monitor horse behaviour (grazing, moving with intent, rolling loafing, drinking) in the montane and subalpine meadows of the USA. It turned out to be efficient in tracking and visualizing animal movements, including periods when direct observation was not possible, e.g. multiple movement patterns associated with grazing that could only be identified by integrating the two methods. This novel system can also be applied to other pack animal species aiding in monitoring and management of domestic animal use and their impacts in natural areas. The methods of behavioural analysis in feral horses are still advancing, making use of modern devices such as drones (Inoue et al., 2019).

## 5 Conclusions

GPS may constitute a valuable source of information about the mobility of free-living horses. This mobility was significantly affected by season (higher mobility in summer and significantly lower in autumn), habitat (higher mobility in the forest and lower in the meadow), and time of the day (higher mobility in the morning and midday and lower in the evening and at night) as well as the interactions among them. Horses changed habitat most frequently in winter and least frequently in autumn. In the present study, the use of the GPS device enabled tracking of horses' mobility also at night, which made the results more complete compared with other similar studies.

## Data Availability

The data are available from the corresponding author upon reasonable request.

## Code availability

Commercial software was used for data analysis (Dell Inc., 2017; Microsoft Inc., 2021). The commercial software is available from the corresponding author upon reasonable request.
